# *trans*-2-(2,5-Dimethoxy-4-iodophenyl)cyclopropylamine and *trans*-2-(2,5-dimethoxy-4-bromophenyl)cyclopropylamine as potent agonists for the 5-HT_2_ receptor family

**DOI:** 10.3762/bjoc.8.194

**Published:** 2012-10-08

**Authors:** Adam Pigott, Stewart Frescas, John D McCorvy, Xi-Ping Huang, Bryan L Roth, David E Nichols

**Affiliations:** 1 Department of Medicinal Chemistry and Molecular Pharmacology, College of Pharmacy, Purdue University, West Lafayette, IN 47907, USA; 2 National Institute of Mental Health, Psychoactive Drug Screening Program, Department of Pharmacology, School of Medicine, University of North Carolina, Chapel Hill, NC 27599, USA

**Keywords:** cyclopropanation, diazomethane, hallucinogen, 5-HT_2A_ agonist, receptor probe, *trans*-2-phenylcyclopropylamines

## Abstract

A strategy to replace the ethylamine side chain of 2,5-dimethoxy-4-iodoamphetamine (DOI, **1a**), and 2,5-dimethoxy-4-bromoamphetamine (DOB, **1b**) with a cyclopropylamine moiety was successful in leading to compounds with high affinity at the 5-HT_2_ family of receptors; and the more potent stereoisomer of the cyclopropane analogues had the expected (−)-(1*R*,2*S*)-configuration. Screening for affinity at various serotonin receptor subtypes, however, revealed that the cyclopropane congeners also had increased affinity at several sites in addition to the 5-HT_2A_ and 5-HT_2B_ receptors. Therefore, at appropriate doses – although (−)-**4** and (−)-**5** may be useful as tools to probe 5-HT_2_ receptor function – one would need to be mindful that their selectivity for 5-HT_2A_ receptors is somewhat less than for DOI itself.

## Introduction

**Figure 1:**
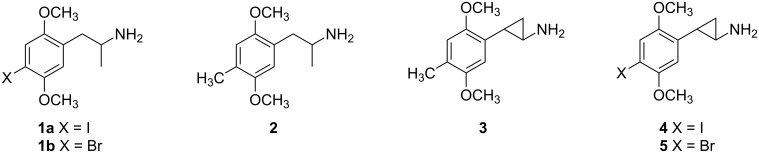
Structures of well-known serotonin 5-HT_2A_ agonists **1a,b**, **2**, and **3**, and compounds **4** and **5** reported in this paper.

Among the molecules that have proven very valuable to neuroscientists studying brain serotonin systems is the substituted phenethylamine derivative 2,5-dimethoxy-4-iodoamphetamine (DOI, **1a**, Figure 1), a potent but nonspecific agonist ligand for serotonin 5-HT_2A_ and 5-HT_2C_ receptors. It is relatively inexpensive and has been widely used throughout the neuroscience community to study behaviors mediated by 5-HT_2_ family receptors. Indeed, as of June 12, 2012, a PubMed search of the terms DOI + 5-HT_2_ yielded 577 hits, spanning from 1984 to the present. Despite the fact that no significant abuse of DOI has been reported, this substance has been scheduled in a number of countries and has been considered for scheduling by the U.S. Drug Enforcement Administration (DEA). Classification as a controlled substance will be a setback to the neuroscience community because it effectively prevents experiments in any laboratory that does not have a proper license for the use of DOI. To illustrate this point, a related compound – bromo congener **1b** (DOB) – presently is a controlled substance and only 52 hits for DOB + 5-HT_2_ were obtained from PubMed, compared with 577 for DOI over the same period of time.

Anticipating the potential need for a substance to replace DOI as a research tool, we sought to identify a molecule that might have pharmacological properties which are identical, or at least very similar to those of **1a**. We had previously characterized the cyclopropane analogue of a hallucinogenic amphetamine known as DOM (**2**) and had shown that **3** (DMCPA) had high potency both in vitro and in vivo [1–3]. We thus considered whether the cyclopropane analogues **4** and **5** might be useful research tools. Accordingly, this report details the synthesis of racemic *trans*-1-(2,5-dimethoxy-4-iodophenyl)-2-aminocyclopropane (**4**) and its bromo homolog **5**, the resolution of **4** into its (−)-(1*R*,2*S*)-enantiomer, as well as the resolution of the cyclopropane carboxylic acid precursor and subsequent bromination to provide both enantiomers of **5**.

## Results and Discussion

Racemic **4** and **5** were compared in radioligand competition assays against radiolabeled antagonists defined at the human 5-HT_2A_ and 5-HT_2C_ receptors and compared with racemic **1a** and **1b**. The results are shown in Table 1. As can be seen, the cyclopropane analogues **4** and **5** showed affinities for the 5-HT_2A_ receptor 5–6-fold greater than **1a** and **1b**. Affinities at the 5-HT_2C_ receptor were about two-fold higher than for **1a** and **1b**.

**Table 1:** Affinity values (*K*_i_ in nM) at human 5-HT_2A_ and 5-HT_2C_ receptors. All values represent mean and SEM from at least three independent experiments.

	^3^H-ketanserin	^3^H-mesulergine

Compound	5-HT_2A_ *K*_i_ in (nM)	5-HT_2C_ *K*_i_ in (nM)

(±)-**1a**	7.6 ± 0.9	35 ± 6
(±)-**1b**	8.9 ± 0.5	31 ± 5
(±)-**4**	1.5 ± 0.1	17 ± 3
(±)-**5**	1.4 ± 0.3	7.5 ± 1.1

The more potent (−)-enantiomers were then tested for functional potency using a calcium release assay. The EC_50_ values and maximal effect at the 5-HT_2A_ receptor were virtually identical for **1a** and **4**, and for **1b** and **5** (Table 2).

**Table 2:** Potency and percent max values for calcium release at 5-HT_2A_ and 5-HT_2C_ receptors. All values represent mean and SEM from at least three independent experiments.

	5-HT_2A_		5-HT_2C_	

Compound	EC_50_ (nM)	%max	EC_50_ (nM)	%max

(−)-**1a**	3.3 ± 0.7	87 ± 1	8.7 ± 0.2	50 ± 5
(−)-**1b**	5.8 ± 1.3	75 ± 7	28 ± 4	59 ± 7
(−)-**4**	2.0 ± 0.3	89 ± 4	21 ± 4	63 ± 6
(−)-**5**	6.3 ± 1.6	76 ± 10	32 ± 8	77 ± 6

At the 5-HT_2C_ receptor **1a** was the most potent, with an EC_50_ that was about three times lower than for **1b**, **4**, or **5**. In functional assays, therefore, the cyclopropane analogues **4** and **5** compared to **1a** or **1b** appeared as potent and had a similar degree of maximal stimulation at each of the respective 5-HT_2_ receptors.

We then carried out a broader screen of **4** and **5** for affinities at a range of other 5-HT receptor isoforms (Table 3). Their affinities at other 5-HT receptors, however, were higher than for **1a**. In particular, the introduction of the cyclopropane appears to increase significantly affinities at the 5-HT_1A_, 5HT_1B_, and 5-HT_1D_ receptors. In that regard, although (−)-**4** and (−)-**5** have affinities at the 5-HT_2A_ receptor somewhat higher than **1a**, their selectivity over the 5-HT_1A_ receptor is less than 100-fold. As shown in Table 1, both **4** and **5** are extremely potent ligands in vitro. Furthermore, as anticipated, it was the (−)-enantiomers that proved to have highest affinity. We included (+)-**5** in Table 3 simply to illustrate the difference in affinity between the two enantiomers. We assume that the final compounds have the (−)-(1*R*,2*S*) and (+)-(1*S*,2*R*) absolute configurations based on our earlier work establishing the absolute configuration of **3** [2], and the fact that substitutions at the 4-position of the aromatic ring in chiral substituted amphetamines do not change the sign of optical rotation [4]. The biological data are consistent with those configuration assignments.

**Table 3:** Affinity values (*K*_i_ in nM) at selected serotonin receptor isoforms.

Cmpd	5-HT_1A_	5-HT_1B_	5-HT_1D_	5-HT_1E_	5-HT_2A_	5-HT_2B_	5-HT_2C_	5-HT_6_	5-HT_7_

(±)**-1a**	<50%^a^	<50%^a^	<50%^a^	1090	9	3	19	1380	850
(±)**-4**	410	290	535	1660	9	10	17	100	580
(−)**-4**	150	230	90	1380	2.4	6	7.4	70	260
**(+)-5**	210	<50%^a^	<50%^a^	<50%^a^	540	20	130	NA	170
**(−)-5**	220	375	390	890	3	4	9	45	120

^a^<50% displacement at 10^−6^ M.

### Chemistry

**Scheme 1:**
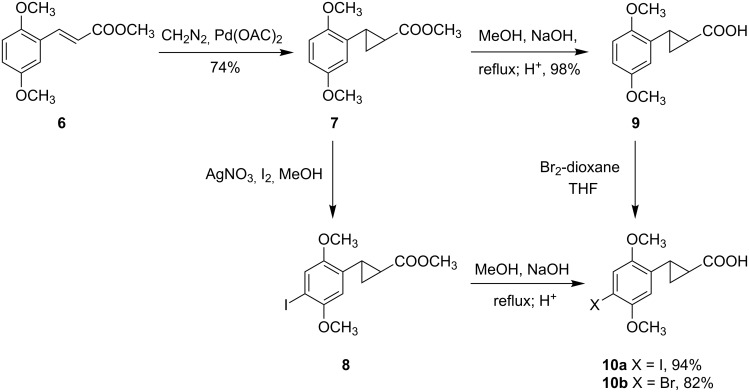
Synthesis of arylcyclopropane carboxylic acids from the corresponding cinnamic acids, followed by halogenation.

**Scheme 2:**
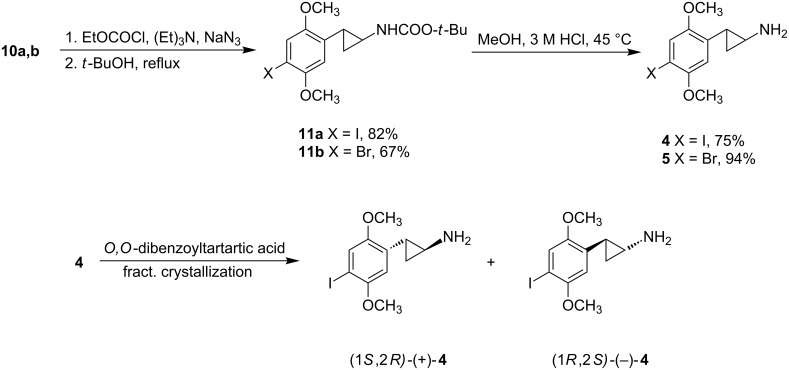
Conversion of arylcyclopropane carboxylic acids **10a,b** to the amines **4** and **5**, and chemical resolution of **4** into its enantiomers.

We reasoned that a palladium-mediated cyclopropanation of the corresponding cinnamic acids would provide the required cyclopropanecarboxylic acid (Scheme 1); which could be readily converted to the amine by a Curtius type rearrangement (Scheme 2). In our previous synthesis [2] we had employed an *N*-carbobenzoxy intermediate, followed by catalytic debenzylation over Pd(C); but those conditions would lead to dehalogenation in the present series, so we instead employed acid-catalyzed removal of a BOC protecting group (Scheme 2).

Thus, we first prepared 2-(2,5-dimethoxyphenyl)cyclopropanecarboxylic acid methyl ester (**7**) from the corresponding cinnamic ester **6** [5], followed by I_2_/AgNO_3_ iodination (Scheme 1), and base hydrolysis of the resulting ester to provide iodo acid **10a**. Hydrolysis of ester **7** followed by bromination of acid **9** using Br_2_-dioxane complex gave a good yield of bromo acid **10b**.

These acids were readily converted to their isocyanates using the Weinstock modification of the Curtius rearrangement [6]. Those isocyanates were heated with *tert*-butanol to afford the corresponding carbamates **11a** and **11b** (Scheme 2). A brief treatment of these with 3 M HCl at 45 °C cleanly affected N-deprotection and afforded the desired final amines **4** and **5**.

The stereochemistry of the more potent enantiomer of DOI is (−)-(*R*) [7], and of **3** is (−)-(1*R*,2*S*) [2]. We therefore undertook the resolution of the enantiomers of DOI by fractional crystallization of the diastereomeric salts prepared with di-*O,O*-benzoyltartaric acid. Unfortunately, we discovered that heating solutions of the dibenzoyltartrate salt of **4** in EtOH or iPrOH led to nearly complete decomposition, presumably through a cyclopropane ring-opening pathway. The apparent need to recrystallize the *O*,*O*-dibenzoyltartrate salts from a nonprotic solvent led us to employ warm acetone, which proved satisfactory. The resolution went well, achieving constant optical rotation after only three crystallizations. The salt was converted to the free base, which was dissolved in dry Et_2_O, followed by addition of the stoichiometric amount of ethereal HCl. The salt precipitated out of solution and could be used directly for pharmacological experiments. Attempts to recrystallize the HCl salt from protic solvents also led to nearly complete decomposition, although salts of bromo compound **5** appeared somewhat more stable.

**Scheme 3:**
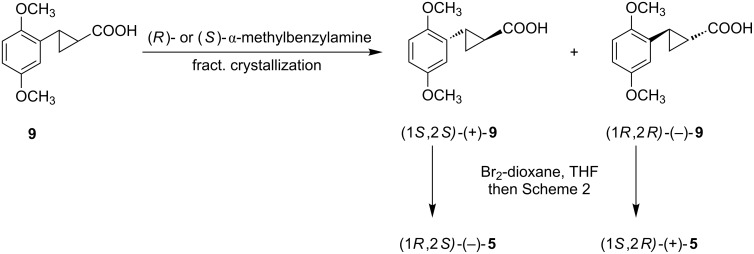
Chemical resolution of arylcyclopropane carboxylic acid **9** followed by bromination.

We then followed a more efficient divergent approach to obtain the enantiomers of **5** that employed resolution of the cyclopropane carboxylic acid, followed by bromination, and then conversion to the cyclopropylamine (Scheme 3). We are aware that the use of chiral auxiliaries in the cyclopropanation step could directly afford the chiral cyclopropane acids [8], but time and resources did not allow us to pursue that approach.

## Conclusion

In conclusion, our strategy to replace the ethylamine side chain of **1a** (or **1b**) with a cyclopropylamine moiety was successful in leading to compounds with high affinity at the 5-HT_2_ family of receptors; and the more potent stereoisomer of the cyclopropane analogues had the expected (−)-1*R*,2*S*-configuration. However, at appropriate doses, although (−)-**4** and (−)-**5** may be useful as tools to probe 5-HT_2_ receptor function, one would also need to be mindful that their selectivity for 5-HT_2A_ over 5-HT_1A_ is only about 70-fold.

The most efficient approach appears to be the synthesis of the chiral cyclopropane carboxylic acids, followed by derivatization at the 4-position. This approach would be most appealing if a chiral auxiliary was used in the cyclopropanation step [8]. We also note that compound **4** was less stable than **5** under recrystallization conditions, an instability we did not observe during our earlier work with **3**. We have observed even greater instability in 2-(indol-3-yl)cyclopropylamines [8–9], suggesting that electron “excessive” π-systems, or the ability to “donate” electrons through resonance (i.e. Br and I), leads to cyclopropane ring instability in 2-arylcyclopropylamines.

## Supporting Information

File 1 Experimental details for all new compounds as well as the pharmacological methods used to measure receptor affinity and functional activity.
