# Supplementary material for: trans-2-(2,5-Dimethoxy-4-iodophenyl)cyclopropylamine and trans-2-(2,5-dimethoxy-4-bromophenyl)cyclopropylamine as potent agonists for the 5-HT2 receptor family
Source: Beilstein J Org Chem. 2012 Oct 8;8:1705–9. doi: 10.3762/bjoc.8.194 (PMC3511003; doi:10.3762/bjoc.8.194)
Supplement: File 1 — Experimental details for all new compounds as well as the pharmacological methods used to measure receptor affinity and functional activity. [file Beilstein_J_Org_Chem-08-1705-s001.pdf]

Supporting Information

for

trans-2-(2,5-Dimethoxy-4-iodophenyl)cyclopropylamine and *trans*-2-(2,5-dimethoxy-4-bromophenyl)cyclopropylamine as potent agonists for the 5-HT$_2$ receptor family

Adam Pigott[1], Stewart Frescas[1], John D. McCorvy[1], Xi-Ping Huang[2], Bryan L. Roth[2] and David E. Nichols*[,§,1]

Address: [1]Department of Medicinal Chemistry and Molecular Pharmacology, College of Pharmacy, Purdue University, West Lafayette, IN 47907 and [2]National Institute of Mental Health, Psychoactive Drug Screening Program, Department of Pharmacology, School of Medicine, University of North Carolina, Chapel Hill, NC 27599

Email: David E. Nichols[*] - drdave@purdue.edu

*Corresponding author

[§]Tel 765-494-1461; fax 765 494-1414

Experimental details for all new compounds as well as the pharmacological methods used to measure receptor affinity and functional activity

Chemistry: General methods

All reagents were commercially available (Aldrich, Alfa Aesar) and were used without further purification unless otherwise indicated. Dry THF was distilled immediately before use from benzophenone-sodium under argon. Column chromatography was carried out using SiliCycle SiliaFlash P60 silica gel (230–400 mesh). J. T. Baker flexible thin layer chromatography sheets (silica gel IB2-F) were used to the monitor reaction progress. Melting points were determined using a Mel-Temp apparatus and are reported as uncorrected values. Optical rotations were obtained at 25 °C on a Rudolph Autopol 1 polarimeter. ^{1}H NMR spectra were recorded using a 300 MHz Bruker ARX300 NMR spectrometer or 500 MHz Bruker DRX500 NMR spectrometer, as noted. Chemical shifts are reported in δ values (ppm) relative to an internal reference (0.03%, v/v) of tetramethylsilane (TMS) in $CDCl_3$, except where noted. Abbreviations used to report NMR peaks are as follows: bs = broad singlet, d = doublet, dd = doublet of doublets, m = multiplet, q = quartet, s = singlet, t = triplet. Electrospray ionization MS analyses were carried out on a FinniganMAT LCQ Classic System. Elemental analyses were performed by the Purdue University Microanalysis Laboratory or by Midwest Microlabs and were within ±0.4% of calculated values. All reactions were carried out under an argon atmosphere, unless noted otherwise.

***trans*-Methyl 2-(2,5-dimethoxyphenyl)cyclopropanecarboxylate (7):** Diazomethane was generated using an Aldrich Mini Diazald apparatus. Diazald (*N*-methyl-nitroso-*p*-toluenesulfonamide, 10.0 g, 46.6 mmol) in 90 mL Et_2O was added dropwise to a mixture containing 5.0 g (89 mmol) KOH in 8 mL water, 16 mL Et_2O, and 25 mL 2-(2-ethoxyethoxy)ethanol which was warmed in a 60 °C oil bath. The diazomethane/ether distillate

was collected with a dry ice/acetone coldfinger condenser and the distillate was added dropwise to a stirred solution of 6.55 g (29.5 mmol) methyl 2,5-dimethoxycinnamate [1] and 32 mg (0.148 mmol) $Pd(OAc)_2$ in CH_2Cl_2/ether (100/50 mL) chilled to −15 °C in an ice/salt bath. The reaction was stirred at −15 °C for 30 min after complete addition of the diazomethane, followed by stirring at room temperature for one hour. Excess diazomethane was quenched with acetic acid, the resulting mixture was washed with a saturated $NaHCO_3$, brine, and dried over $MgSO_4$. After filtration, the organic solvent was removed by rotary evaporation. The residue, which contained some unreacted starting material, was recrystallized from Et_2O. The crude tan crystals were recrystallized a second time to provide 5.08 g (74.2%) of white crystalline product. An analytical sample was prepared by sublimation at 50 °C/0.05 torr; mp 52 °C, ^1H NMR (300 MHz, $CDCl_3$) δ 6.77 (d, J = 8.41Hz, 1H), 6.69 (dd, J = 2.19, 8.41 Hz, 1H), 6.45 (d, J = 2.19 Hz, 1H), 3.79 (s, 3H), 3.75 (s, 3H), 3.73 (s, 3H), 2.78–2.70 (m, 1H), 1.90–1.82 (m, 1H), 1.61–1.53 (m, 1H); MS (ESI) 237 $[M + H]^+$.

***trans*-Methyl 2-(4-iodo-2,5-dimethoxyphenyl)cyclopropanecarboxylate (8):** A solution of **7** (12.15 g, 51.5 mmol), $AgNO_3$ (9.57 g 56.6 mmol) and iodine (14.32 g, 56.6 mmol) in methanol was stirred under argon at room temperature overnight. The yellow precipitate was removed by filtration and washed on the filter with EtOAc. The filtrate was treated dropwise with saturated $NaHSO_3$ to reduce the remaining iodine. The solvent was then removed and the resulting solid was suspended in water and collected by filtration to afford the iodo ester as a pale yellow solid (17.42 g, 94%); mp 98–100 °C, which was pure enough to be carried forward in the synthesis. ^1H NMR (300 MHz, $CDCl_3$) δ 7.19 (s, 1H), 6.38 (s, 1H,), 3.78 (s, 3H), 3.77 (s, 3H), 3.71 (s, 3H), 2.69–2.65 (m, 1H), 1.87–1.82 (m, 1H), 1.58–1.52 (m, 1H), 1.32–1.27 (m, 1H); ^{13}C NMR

(75 MHz, $CDCl_3$) δ 173.9 (C), 153.1 (C), 152.5 (C), 129.5 (C), 121.6 (CH), 109.5 (CH), 82.8 (C), 57.1 (CH_3), 56.2 (CH_3), 51.9 (CH_3), 22.4 (CH), 21.4 (CH), 15.7 (CH_2); MS (ESI) 363.03 $[M + H]^+$.

***trans*-2-(2,5-Dimethoxyphenyl)cyclopropanecarboxylic acid (9):** The pure ester **7** (23.63 g, 100 mmol) was placed in a flask containing 100 mL methanol. NaOH (40 mL of 50% aqueous solution) was then added to the stirring mixture. The reaction was stirred at reflux overnight. The clear solution was cooled, acidified to pH 2 with 0.5 M HCl, the mixture was concentrated by rotary evaporation and 150 mL CH_2Cl_2 was added to dissolve the carboxylic acid. The aqueous phase was separated and extracted with 3 x 20 mL CH_2Cl_2 and the combined organic extracts were washed with H_2O, brine, dried over $MgSO_4$, then filtered and concentrated in vacuo. The crude crystals were recrystallized from EtOH to afford the title compound as a white crystalline solid (21.77, 98%); mp 101 °C. ^1H NMR (300 MHz, $CDCl_3$) δ 6.89 (d, *J* = 8.85 Hz, 1H), 6.72 (dd, *J* = 3.24, 10.2 Hz, 1H), 6.48 (d, *J* = 3.35 Hz, 1H), 3.81 (s, 3H), 3.75 (s, 3H), 2.84–2.76 (m, 1H), 1.88–1.80 (m, 1H), 1.66–1.58 (m, 1H), 1.44–1.36 (m, 1H); MS (ESI) 221 $[M – H]^–$.

***trans*-2-(4-Iodo-2,5-dimethoxyphenyl)cyclopropanecarboxylic acid (10a):** To a stirred solution of 17.41 g (48 mmol) **8** in 200 mL methanol was added 40 mL aqueous 2 M NaOH and the resulting solution was heated at reflux overnight. Then, the reaction was cooled, acidified with 1 M HCl, and the methanol was removed in vacuo. The aqueous phase was extracted with 3 x 60 mL CH_2Cl_2 and the combined organic extracts were washed with brine, dried over Na_2SO_4, and concentrated in vacuo to provide the title compound as a white solid (17.02 g, 97%); mp 162–163 °C; ^1H NMR (300 MHz, MeOD) δ 7.26 (s, 1H), 6.52 (s, 1H), 3.78 (s, 3H),

3.76 s, 3H), 2.61–2.57 m, (1H), 1.77–1.72 (m, 1H), 1.47–1.37 (m, 2H); ^{13}C NMR (75 MHz, MeOD) δ 177.3 (C), 154.6 (C), 154.2 (C), 131.0 (C), 122.9 (CH), 110.5 (CH), 83.4 (C), 57.5 (CH_3), 56.8 (CH_3), 23.7 (CH), 22.3 (CH), 15.8 (CH_2); MS (ESI) 349.24 $[M + H]^+$.

***trans*-*tert*-Butyl 2-(4-iodo-2,5-dimethoxyphenyl)cyclopropylcarbamate (11a):** A mixture of 8.95 g (25 mmol) **10a**, 5.0 mL (36 mmol) triethylamine, and 3.70 mL (38 mmol) ethyl chloroformate in 150 mL dry acetone was stirred at −10 °C. After 2.5 h, a solution of 2.84 g (43.7 mmol) NaN_3 in 10 mL H_2O was added. The stirring was stopped after an additional 2 h. The resulting suspension was poured into 100 mL cold H_2O and was extracted with 4 x 25 mL toluene. The combined organic layers were dried over Na_2SO_4 and concentrated to about 50% volume to remove remaining traces of H_2O. The resulting solution was heated at 90 °C until nitrogen evolution ceased (3 h), and was then concentrated by rotary evaporation. The resulting isocyanate was dissolved in 50 mL dry *t*-BuOH, and the solution was heated at reflux overnight. The reaction mixture was concentrated, and the crude product was purified by flash chromatography (9:1 hexanes/EtOAc) to give the title compound as a pale yellow solid. (9.22 g, 85%); mp 123–124°C; ^{1}H NMR (300 MHz, $CDCl_3$) δ 7.16 (s, 1H), 6.36 (s, 1H), 4.98 (bs, 1H), 3.77 (s, 3H), 3.76 (s, 3H), 2.72 (bs, 1H), 2.19–2.13 (m, 1H), 1.42 (s, 9H) 1.17–1.09 (m, 2H); ^{13}C NMR (75 MHz, $CDCl_3$) δ 156.2 (C), 152.9 (C), 152.5 (C), 130.5 (C), 121.5 (CH), 109.5 (CH), 81.9 (C), 79.5 (C), 57.0 (CH_3), 56.3 (CH_3), 31.8 (CH), 28.3 (CH_3), 19.2 (CH), 16.2 (CH_2); MS (ESI) 441.58 $[M + Na]^+$.

(±)-*trans*-2-(4-Iodo-2,5-dimethoxyphenyl)cyclopropylamine hydrochloride (4): To a solution of 9.20 g (21.9 mmol) **11a** in 900 mL MeOH was added 50 mL aqueous 3 M HCl. The reaction

was warmed to 45 °C and stirred overnight. Then, it was diluted with 200 mL aqueous NH_4Cl and the MeOH was removed in vacuo. The acidic aqueous phase was washed with 3 x 50 mL CH_2Cl_2 and then basified with $NaHCO_3$ and extracted with 3 x 50 mL CH_2Cl_2. The organic extracts were combined, dried over $NaSO_4$, filtered, and concentrated to give 2-(4-iodo-2,5-dimethoxyphenyl)cyclopropanamine as a colorless oil (5.85 g, 75%), which was then converted to the hydrochloride salt by dissolving the base in dry Et_2O and adding the stoichiometric amount of ethereal HCl to produce a white crystalline solid; mp 231–232 °C(dec); ^{1}H NMR (300 MHz, D_2O) δ 7.28 (s, 1H), 6.50 (s, 1H), 3.75 (s, 3H), 3.71 (s, 3H), 2.83–2.78 (m, 1H), 2.46–2.39 (m, 1H), 1.39–1.32 (m, 2H); ^{13}C NMR (75 MHz, D_2O) δ 153.3 (C), 152.6 (C), 128.5 (C), 122.7 (CH), 111.4 (CH), 83.4 (C), 57.7 (CH_3), 57.1(CH_3), 30.4 (CH), 17.1 (CH), 11.5 (CH_2); MS (ESI) 320.56 $[M + H]^+$.

Resolution of (−)-*trans*-(1*R*,2*S*)-2-(4-Iodo-2,5-dimethoxyphenyl)cyclopropylamine hydrochloride ((−)-4): A solution of 860 mg (2.7 mmol) of the free base **4** was dissolved in 50 mL of Et_2O. A solution of 1.08 g (2.7 mmol) of (+)-*O*,*O*'dibenzoyl-D-tartaric acid in 50 mL of Et_2O was then added, whereupon the diastereomeric salt immediately precipitated. The salt was collected by filtration, washed on the filter with Et_2O and air-dried to afford a quant yield of salt as an off-white powder. The initial rotation of this material was $[\alpha]_D$ +58° (*c* 0.5, MeOH) with mp 157 °C. The salt was dissolved in a minimum volume of warm acetone and allowed to stand overnight at room temperature, and then at 0 °C for 24 h. The resulting crystals were collected by filtration and had $[\alpha]_D$ +54° (*c* 0.5, MeOH) and mp 161 °C. The crystallization process was repeated two more times, at which point the rotation had ceased to change, with a final $[\alpha]_D$ +22° (*c* 0.5, MeOH) and a mp of 163–164 °C. The salt was dissolved in H_2O and excess 2 M NaOH was

added to liberate the free base, which was extracted into 3 x 20 mL Et_2O. The combined organic extracts were dried over $MgSO_4$ and then filtered. Addition of a solution of anhyd. HCl–Et_2O precipitated the HCl salt as fine white crystals with $[\alpha]_D$ –47.9° (*c* 0.5, MeOH) and mp 231–232 ^oC (dec). The salt was not further purified because of its relative instability in warm protic solvents. The ^1H NMR was identical to that of the racemic material prepared earlier.

Resolution of *trans*-2-(2,5-dimethoxyphenyl)cyclopropanecarboxylic acid (9): Racemic **9** (22.22 g, 100 mmol) was dissolved in 200 mL Et_2O. (*S*)-(+)-α-methylbenzylamine (12.12 g, 100 mmol) in 50 mL Et_2O was added to the stirred solution whereupon a voluminous white precipitate formed. The mixture was stirred for 1 h and filtered, providing 34.42 g of the crude salt, that had $[\alpha]_D$ −2.0° (*c* 0.5, MeOH) and mp 98–100 °C. The salt was recrystallized three times from acetonitrile to provide 13.83 g (40.2%) of the pure diastereomeric salt: mp 134–137 °C; $[\alpha]_D$ −131° (*c* 0.5, MeOH). The salt was dissolved in H_2O and the carboxylic acid was liberated by careful acidification to pH 2 with 0.5 M HCl. CH_2Cl_2 was added to dissolve the solids, the layers were separated, and the aqueous phase was extracted with 3 x 50 mL CH_2Cl_2. The organic solutions were combined, dried over $MgSO_4$, filtered, and the solvent removed by rotary evaporation. The residue was recrystallized from ethanol, providing 8.58 g (38.6%) of (1*S*,2*S*)-(+)-(**9**) as a white crystalline solid, mp 101 °C, $[\alpha]_D$ +191° (*c* 0.5, MeOH) ^1H NMR and MS data as described for (±)-**9**. Mother liquors enriched in the (1*R*,2*R*)-isomer from multiple resolutions were combined, converted to the free acid, and treated with (−)-(*R*)-α-methylbenzylamine to form the crude salt, which after three recrystallizations from acetonitrile provided the pure diastereomeric salt: mp 135–137 °C; $[\alpha]_D$ +132° (*c* 0.5, MeOH). The free (−)-(1*R*,2*R*)-carboxylic acid was obtained as described above: mp 101 °C, $[\alpha]_D$ -191° (*c* 0.5, MeOH).

(−)-(1*R*,2*R*)-*trans*-2-(4-Bromo-2,5-dimethoxyphenyl)cyclopropanecarboxylic acid ((−)-10b)): (−)-(1*R*,2*R*)-**9** (5.0 g, 22.5 mmol) was dissolved in 150 mL of THF in a stirred flask cooled in an ice bath. Br_2–dioxane complex (120 mL, 0.25M, 1.4 equiv) was slowly added dropwise. The reaction was stirred at 0 °C for 3 h, when NMR analysis showed that the reaction had gone to completion. The reaction was quenched by addition of an aq solution containing 2 M NaOAc/2 M sodium thiosulfate, which changed the color from orange to clear. EtOAc (100 mL) was added, the aqueous layer was separated, washed with ethyl acetate, and the combined organic solutions were washed with brine and dried over $MgSO_4$. After filtration, the solvent was removed by rotary evaporation. The crude tan crystals were recrystallized from EtOH to provide 5.50 g (82%) of the title compound as a white crystalline solid; mp 179 °C, $[\alpha]_D$ −169° (*c* 0.5, MeOH); ^{1}H NMR (300 MHz, $CDCl_3$) δ 7.03 (s, 1H), 6.50 (s, 1H), 3.83 (s, 3H), 3.81 (s, 3H), 2.75–2.71 (m, 1H), 1.87–1.83 (m, 1H), 1.66–1.63 (m, 1H), 1.43–1.38 (m, 1H); ^{13}C NMR (125 MHz, $CDCl_3$) δ 179.6 (C), 152.8 (C), 149.9 (C), 127.8 (C), 115.9 (CH), 111.0 (CH), 109.6 (C), 57.0 (CH_3), 56.2(CH_3), 22.4 (CH), 22.2 (CH), 16.1 (CH_2); MS (ESI) 299.35, 301.64 $[M – H]^-$.

(+)-(1*S*,2*S*)-*trans*-2-(4-Bromo-2,5-dimethoxyphenyl)cyclopropanecarboxylic acid ((+)-10b)): Following the procedure given for the preparation of (−)-**10b** from (+)-**9** (2.87 g, 12.9 mmol) provided 3.10 g (79.8%) (+)-**10b**; mp 178 °C, $[\alpha]_D$ +166° (*c* 0.5, MeOH) with NMR and MS data identical to (−)-**10b**.

(−)-(1*R*,2*S*)-*N*-*tert*-Butyloxycarbonyl-2-(4-bromo-2,5-dimethoxyphenyl)cyclopropane-carboxamide ((−)-11b)): A mixture of (−)-(1*R*,2*R*)-**10b** (4.19, 13.9 mmol), triethylamine (1.97 g, 19.5 mmol), and ethyl chloroformate (2.26 g, 20.8 mmol) in 80 mL dry acetone was

stirred at −10 °C for 2.5 h. A solution of NaN_3 (1.58 g, 24.3 mmol) in 5 mL H_2O was then added. Stirring was continued for additional 2 h. The resulting suspension was poured into 75 mL cold H_2O and extracted with 4 x 25 mL toluene. The combined organic layers were dried over $MgSO_4$, filtered, and concentrated to about 50% volume to remove remaining traces of H_2O. The resulting solution was heated at 90 °C until the evolution of nitrogen ceased (3 h). The reaction was then concentrated by rotary evaporation, the resulting residual isocyanate was dissolved in 25 mL dry *t*-BuOH, and the reaction was heated at reflux overnight. After concentration, the crude product was purified by recrystallization from EtOH to provide the title compound as a fine white powder (3.49 g, 67.5%); mp 138–140 °C, $[\alpha]_D$ −91.4° (*c* 0.5, MeOH); ^{1}H NMR (300 MHz, $CDCl_3$) δ 7.00 (s, 1H), 6.47 (s, 1H), 4.90, (bs, 1H), 3.81 (s, 3H), 3.80 (s, 3H), 2.75–2.71 (m, 1H), 2.19–2.14 (m, 1H), 1.45 (s, 9H), 1.20–1.12 (m, 2H); ^{13}C NMR (125 MHz, $CDCl_3$) δ 156.2 (C), 152.5 (C), 150.0 (C), 129.3 (C), 115.7 (CH), 110.6 (CH), 108.6 (C), 79.6 (C), 56.9 (CH_3), 56.2 (CH_3), 31.8 (CH), 28.3 (CH_3), 19.2 (CH), 16.2 (CH_2); MS (ESI) 393.74, 395.81 $[M + Na]^+$.

(+)-(*1S,2R)-N-tert*-Butyloxycarbonyl-2-(4-bromo-2,5-dimethoxyphenyl)cyclopropane-carboxamide ((+)-11b)): Following the procedure given for the preparation of (−)-**11b** 1.51g, (5.0 mmol) of (+)-**10b** yielded 1.29 g (69.3%); mp 139–140 °C, $[\alpha]_D$ +91.3° (*c* 0.5, MeOH) NMR and MS data as given for (−)-**11b**.

(−)-(1*R*,2*S*)-*trans*-2-(4-Bromo-2,5-dimethoxyphenyl)cyclopropylamine hydrochloride ((−)-4)): To a solution of (−)-**11b** (45 mg, 0.12 mmol) in MeOH (20 mL) was added 1.5 mL aqueous 3 M HCl. The reaction was warmed to 45 °C and stirred overnight. The solution was then diluted

with 20 mL aqueous NH_4Cl and washed with 3 x 10 mL CH_2Cl_2. The aqueous phase was basified with 1 M NaOH and extracted with 3 x 15 mL CH_2Cl_2. The organic extracts were combined and dried over $MgSO_4$, then filtered and concentrated to provide **5** (31 mg, 94%), which was converted to the hydrochloride salt and recrystallized from MeOH/Et_2O; mp 238–239 °C, $[\alpha]_D$ −55.5° (*c* 0.5, MeOH); ^{1}H NMR (300 MHz, D_2O) δ 7.19 (s, 1H), 6.63 (s, 1H), 3.78 (s, 3H), 3.75 (s, 3H), 2.80–2.75 (m, 1H), 2.43–2.36 (m, 1H), 1.39–1.30 (m, 2H); ^{13}C NMR (125 MHz, D_2O) δ 153.2 (C), 149.9 (C), 127.6 (C), 117.0 (CH), 112.7 (CH), 109.7 (C), 57.5 (CH_3), 57.1(CH_3), 30.3 (CH), 16.9 (CH), 11.5 (CH_2); MS (ESI) 271.71, 273.75 $[M + H]^+$.

(+)-(1*S*,2*R*)-*trans*-2-(4-Bromo-2,5-dimethoxyphenyl)cyclopropylamine hydrochloride ((+)-4)): Following the procedure given for the preparation of (−)-**4**, 1.20 g (3.22 mmol) of (+)-**11b** afforded 0.647 g (65.1%); mp 227 °C; $[\alpha]_D$ +53.4° (*c* 0.5, MeOH) with NMR and MS data identical to (−)-**4**.

Pharmacology

Radioligand binding assays

Materials. Radioligands were purchased from Perkin-Elmer Life and Analytical Sciences (Waltham, MA): [^{3}H]-ketanserin (60 Ci/mmol) for 5-HT_{2A} binding and [^{3}H]-mesulergine (84.3 Ci/mmol) for 5-HT_{2C} binding. 5-HT creatinine phosphate was purchased from Sigma-Aldrich Chemical Company (St. Louis, MO).

Cell culture and tissue preparation. All studies used transiently transfected human embryonic kidney (HEK) 293 cells expressing either the human 5-HT_{2A} or 5-HT_{2C} receptors subcloned into

pcDNA3.1. HEK cells were cultured in Dulbecco's Modified Eagle's Medium (DMEM) supplemented with 10% dialyzed fetal bovine serum (Atlanta Biologicals, Lawrenceville, GA), and 100 U/mL penicillin, 100 μg/mL streptomycin, 0.25 μg/mL amphotericin B. Cells were grown at 37 °C in a humidified incubator with 5% CO_2. Calcium phosphate transient transfections were performed in 15 cm tissue culture dishes, with cells grown to approximately 60–70% confluency on the day of transfection and allowed to grow 48 h until membrane preparation for binding assays or for Aequorin calcium release assays.

Competition binding experiments. All drug dilutions were made in binding buffer (50 mM Tris, 4 mM $MgCl_2$, pH 7.4) and performed in 96-well assay tubes containing drug concentrations ranging from 10^{-12} to 10^{-4} M in a final volume of 500 μL. Radioligands used were 1–2 nM [^{3}H]-ketanserin for 5-HT_{2A}, and 2-3 nM [^{3}H]-mesulergine for 5-HT_{2C}. All binding experiments were incubated at 25 °C for one hour and terminated by rapid filtration with a 96-well Packard Filtermate cell harvester with ice cold wash buffer (10 mM Tris, 0.9% NaCl). Filter plates were dried, and 40 μL of Packard Microscint-O was added to each filter well. Radioactivity was counted using a Packard Topcount scintillation counter.

Aequorin calcium release assays. HEK cells expressing the apoaequorin protein were purchased from Perkin-Elmer Life and Analytical Sciences (Waltham, MA). Bovine serum albumin (BSA) medium was prepared using DMEM/Hams F12 without phenol red containing 15 mM HEPES and 0.1% BSA (protease free). Transfected cells were resuspended and transferred to 5 mL centrifuge tubes, and centrifuged for two minutes at 1500 RPM in a clinical centrifuge. The supernatant was decanted, cells were resuspended in 5 mL of BSA medium and

incubated with 5 µM coelenterazine h for three hours in the dark on a rotating plate. Viable cells were counted with trypan blue and cells were diluted using BSA media to 2 x 10^5 cells/mL (10,000 cells per 50 µL) in a foil wrapped 20-mL beaker equipped with a 1.1 cm stir bar. Cells were then incubated with constant stirring for an additional 1 hour until the time of the assay. Luminescence measurements were performed using a Victor Light 1420 Luminescence Counter from Perkin-Elmer. Cells were dispensed (50 µL) using a syringe injector into 96-well Optiplates containing 50 µL of either 100 µM digitonin, BSA buffer for basal, or various concentrations of test drug. Luminescence reads occurred immediately upon dispensing cells into the well and lasted for exactly 40 seconds.

Data analysis. GraphPad Prism 4 software (San Diego, CA) was used to generate nonlinear regression curves for radioligand displacement and calculation of dose-response curves. For competition assays, K_i values were generated from IC_{50} values with hill slopes fixed to −1.0. K_i values were calculated using the Cheng–Prusoff equation utilizing the assay radioligand concentration and the radioligand K_D, which was 1.2 nM for [^{3}H]-ketanserin at h5-HT_{2A} and 2.3 nM for [^{3}H]-mesulergine at h5-HT_{2C}. For aequorin calcium release assays, the area under the curve (AUC) was used for each 40 second read per well to yield counts per second (CPS) x seconds, which was used to generate the EC_{50} and percent max stimulation (%max). The EC_{50} value was generated from variable slope sigmoidal dose-response analysis, and the %max was generated using 10 µM 5-HT.

PDSP screening. Radioligand competition binding assays were carried out by the Psychoactive Drug Screening Program (PDSP, http://pdsp.med.unc.edu) using crude membrane preparations

of HEK293 cells expressing human 5-HT receptors (stable lines or transient transfections). Detailed information about radioligand binding assays is in the PDSP assay protocol book and is available on-line (http://pdsp.med.unc.edu/pdspw/binding.php). Results were analyzed in GraphPad Prism 5.0 and K_i values were obtained from Prism's built-in three-parameter logistic function for one-site competition binding.

References

1. Peterson J. R.; Russell M. E.; Surjasasmita I. B. *J. Chem. Eng. Data* **1988,** *33*, 534–537. doi:0.1021/je00054a042
